# IgG4 Related Disease Presenting With Eosinophilic Pleural Effusion

**DOI:** 10.1002/rcr2.70111

**Published:** 2025-02-15

**Authors:** Ancy Elsa Thomas, Balamugesh Thangakunam, Thomas Kodiatte, Devasahayam Jesudas Christopher

**Affiliations:** ^1^ Department of Pulmonary Medicine Christian Medical College Hospital Vellore India

**Keywords:** eosinophilia, IgG4 related disease, pleural effusion, thoracoscopy

## Abstract

A 56‐year‐old man presented with right‐sided pleuritic chest pain and intermittent cough for 4 months, with a background history of well‐controlled asthma and allergic rhinitis. Chest radiographs revealed right‐sided pleural effusion, the fluid chemistry showed an exudate effusion with negative cytology and abundant eosinophils. Thoracoscopic pleural biopsy showed moderate lymphoplasmacytic chronic inflammation. Immunohistochemistry showed IgG4 staining up to 90 plasma cells/hpf (> 10% diagnostic), and the IgG4: IgG ratio was 40% (> 40% diagnostic), confirming the diagnosis of IgG4‐related disease (IgG4‐RD). Elevated serum IgG4 level (300 mg/dL; normal range: 3–201 mg/dL), further supported the diagnosis. While eosinophilic pleural effusion (EPE) accounts for 5%–16% of all exudative pleural effusions, IgG4‐related pleural disease is an extremely rare cause of EPE.

## Graphical abstract

Pleural disease is uncommon in IgG4‐RD and pleural effusion is even less frequent. Also, IgG4‐RD is an extremely rare cause of eosinophilic pleural effusion. To our knowledge, this is the first report of IgG4‐related eosinophilic pleural effusion from India.

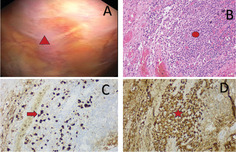

## 1 Introduction

Immunoglobulin G4‐related disease (IgG4‐RD) is an immune‐mediated fibroinflammatory disease that could affect multiple organs characterized by an elevation in serum IgG4 levels and the presence of IgG4‐positive plasma cells in tissues and organs [1]. It could affect several organs, particularly; the pancreas, liver and biliary tree, kidneys, thyroid gland, lungs, and aorta [2]. Up to 50% of patients may experience thoracic involvement, which can manifest as mediastinal lymphadenopathy, lung consolidations, pericarditis, and pleural disease. Pleural involvement is seen in 16% of IgG4‐related diseases, and pleural effusion is even rarer, occurring in only 5% [3]. While eosinophilic pleural effusion (EPE) accounts for 5%–16% of all exudative pleural effusions, IgG4‐related disease is an extremely rare cause of EPE. The diagnosis primarily relies on tissue biopsy and histopathological evaluation, which reveal dense fibrosing pleuritis accompanied by prominent lymphoplasmacytic inflammation, including IgG4‐positive plasma cells [4]. Herein, we are reporting a case of IgG4‐RD, and to our knowledge, this is the first case report of IgG4‐RD with eosinophilic pleural effusion from India.

## 2 Case Report

**FIGURE 1:**
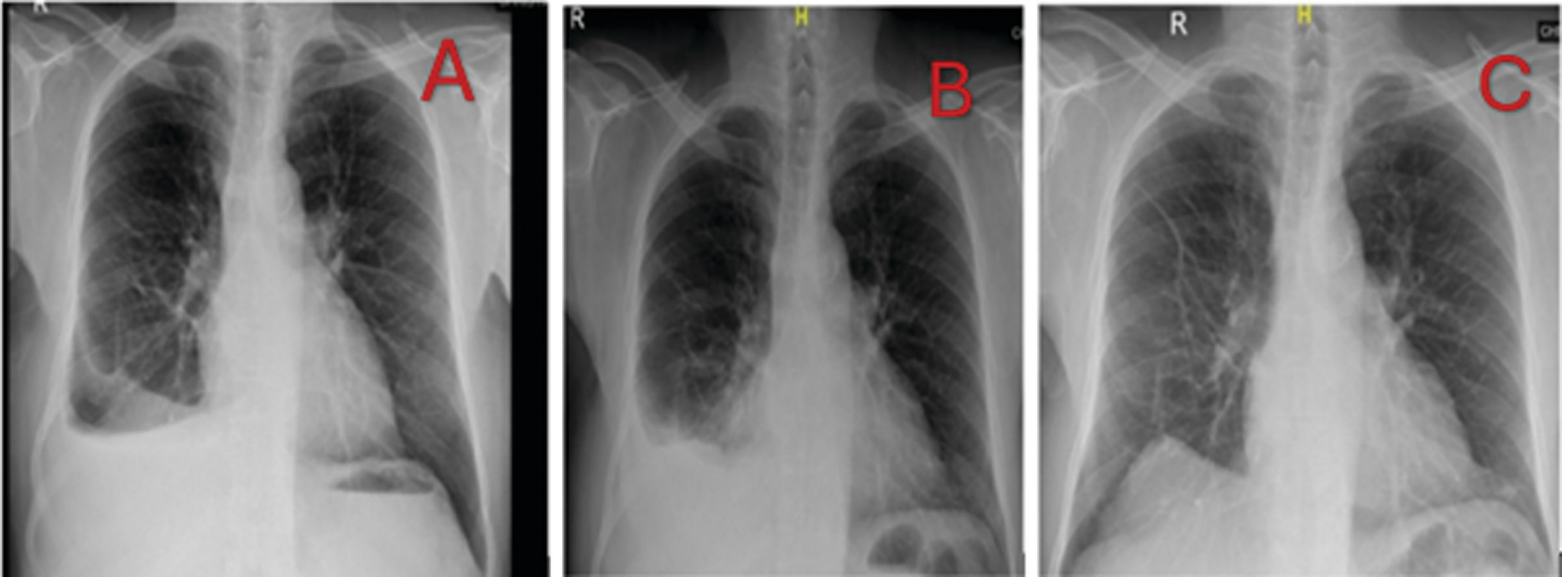
Chest radiographs. (A) Chest radiograph showing right‐sided pleural effusion with non‐homogenous opacities suggesting probable underlying parenchymal changes. (B) Chest radiograph after 2 months showing increase in the right‐sided pleural effusion. (C) Chest radiograph showing resolution of the right‐sided pleural effusion.

A 56‐year‐old male college professor, a non‐smoker, presented with complaints of right pleuritic chest pain and intermittent cough for 4 months. There is no history of fever, loss of weight, or loss of appetite. There was no history of malignancy, autoimmune disorder, or contact with tuberculosis. He was diagnosed with asthma and intermittent allergic rhinitis 3 years ago, and his symptoms were under good control with a combination of fluticasone and formoterol administered through a metered‐dose inhaler. He was evaluated elsewhere and was found to have right‐sided pleural effusion, for which he was prescribed an antibiotic (Amoxicillin+Clavulanic acid), which he was continuing to take when he reported for evaluation. Lab investigations showed peripheral eosinophilia (8%), with an absolute eosinophil count of 664 cells/mm^3^. Renal and Liver function tests including serum albumin were normal. Antineutrophil cytoplasmic antibodies (ANCA) and connective tissue disorder screening tests were negative. Chest radiographs revealed mild right‐sided pleural effusion with non‐homogenous opacities in the parenchyma (Figure 1A). Computed tomography (CT) thorax showed interstitial thickening and a few ground glass opacities in the right lung, which were attributed to resolving infection, but there were no consolidations. Chest ultrasound confirmed hypoechoic fluid collection in the right hemithorax with no septations, extending over one and a half intercostal spaces posteriorly and one intercostal space anteriorly. There was pleural thickening involving parts of the parietal and diaphragmatic pleura. There was no consolidation or B lines in the right lung. Pleural fluid aspiration was performed, and it was a haemorrhagic and eosinophil predominant (80%) exudate, with low adenosine deaminase (13 U/L). Pleural fluid for Xpert‐MTB/RIF did not detect *Mycobacterium tuberculosis* , and cytology was negative for malignant cells. He fulfilled the criteria for the diagnosis of eosinophilic pleural effusion (EPE) (> 10% of eosinophils) [5]. Since he had already commenced antibiotics for a presumptive diagnosis of parapneumonic effusion; at that time we opted to complete the treatment and keep him under close follow‐up.

**FIGURE 2:**
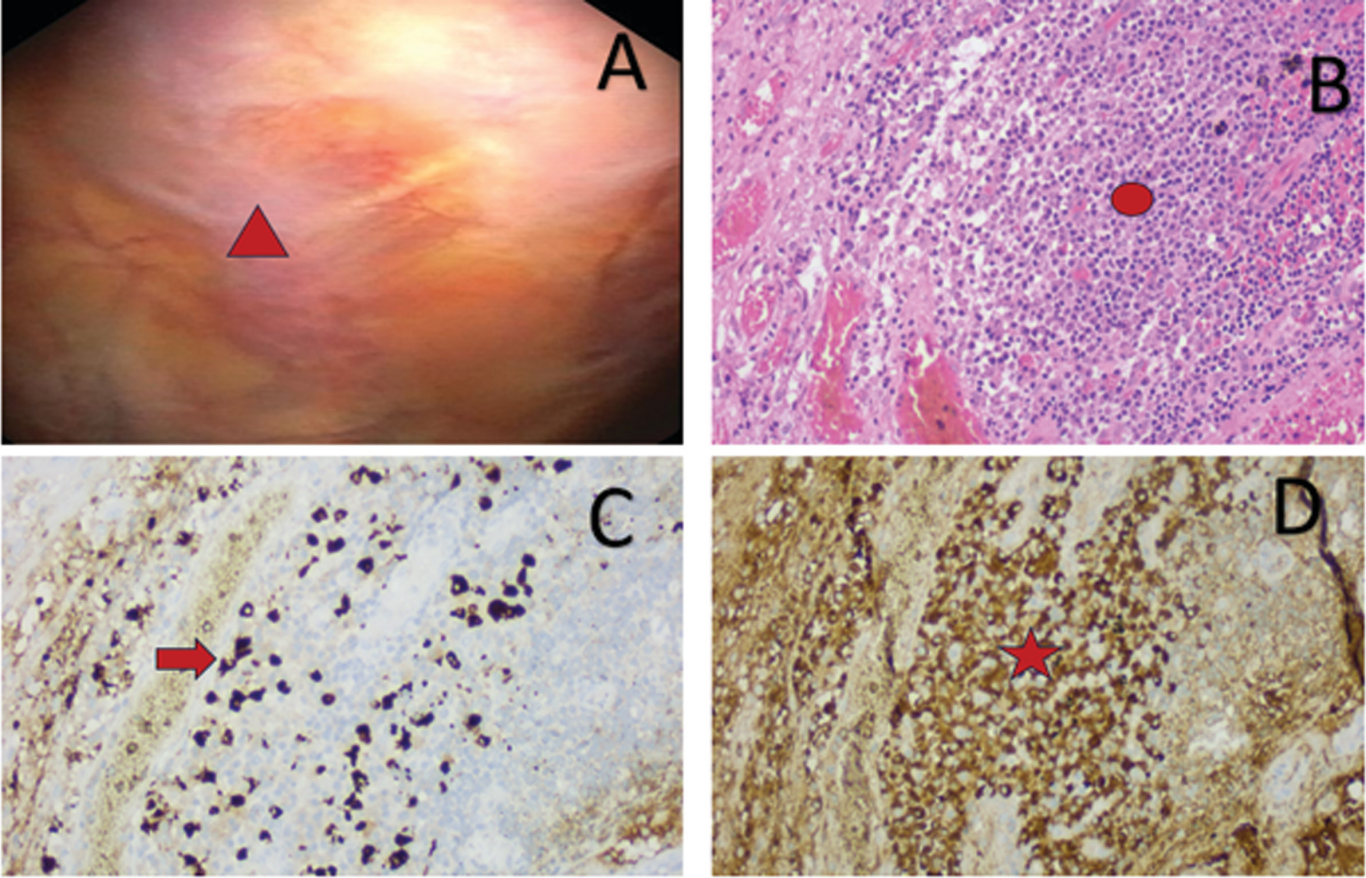
Pleural pathology—thoracoscopic and photomicrographic appearance. (A) Thoracoscopic picture showing whitish plaques (triangle) on the costal pleura. (B) Photomicrograph displaying many sheets of plasma cells (circle), H&E. (C) Photomicrograph displaying many IgG4 positive plasma cells (arrow), IgG4 IHC. (D) Photomicrograph displaying many IgG positive plasma cells (star), IgG IHC.

Two months later, the patient continued to have pleuritic chest pain, and the follow‐up chest radiograph showed an increase in the size of the right‐sided pleural effusion (Figure 1B). Ultrasonography confirmed the increase in pleural fluid, but it was still inadequate to safely perform thoracoscopy. Therefore, iatrogenic pneumothorax was created using a Boutin needle, and Medical thoracoscopy was done. It revealed pleural thickening and whitish plaques in the costal pleura, which were biopsied. There were no nodules or adhesions. (Figure 2A). The pleural tissue was negative for parasites.

The pleural biopsy showed moderate lymphoplasmacytic chronic inflammation (Figure 2B) with no specific diagnosis. Immunohistochemistry (Figure 2C,D) showed IgG4 positive staining up to 90 plasma cells/hpf (> 10/hpf diagnostic), and the IgG4:IgG ratio was 40% (> 40% diagnostic) [4]. The level of his serum IgG4 was also high at 300 mg/dL (normal range: 3–201 mg/dL). A diagnosis of IgG4‐RD involving pleura as the cause of pleural effusion was made. Since the patient had no systemic symptoms and had normal blood tests, it was felt that involvement of other organs was unlikely.

Steroid treatment was commenced with oral prednisolone at a dosage of 0.5 mg/kg. Follow‐up chest radiograph (after 1 month) showed complete resolution of the right‐sided pleural effusion (Figure 1C). He remained well on 1 year follow‐up, at which point steroid treatment was stopped in discussion with the patient. When he was reviewed 6 months after the cessation of steroid treatment, he remained well, and there was no recurrence of the pleural effusion.

## 3 Discussion

This case demonstrates an uncommon presentation of IgG4‐RD in India; the patient presented with few lung opacities and the main problem was a non‐resolving pleural effusion. He was initially treated as a case of parapneumonic effusion, though the pleural fluid analysis revealed an EPE. Persistent pleuritic pain and increasing pleural effusion necessitated medical thoracoscopy and the pleural biopsy sample confirmed IgG4‐related disease. Treatment with oral prednisolone led to the complete resolution of pleural effusion, and the patient remained well without recurrence 6 months after discontinuing steroid therapy.

Formerly referred to as Mikulicz's illness, IgG4‐RD was initially observed in cases of autoimmune pancreatitis and inflammation of salivary and lacrimal glands. Approximately 40% of patients exhibit thoracic involvement [6]. This is primarily observed in middle‐aged to elderly males (mean age: 63.5 ± 14.7) [7]. Pleural involvement is characterized by the presence of nodules and pleural thickening and in a Chinese cohort, 16.1% had pleural nodules or thickening, but pleural effusion was less common, occurring in approximately 4.6% of the cases [3]. Thus, pleural effusion is uncommon in IgG4‐RD, which in turn is an uncommon cause of EPE. The common causes of EPE are malignancies, infections, trauma, and parasites [4, 8]. Unilateral EPE caused by IgG4‐RD is rarely reported [9].

IgG4‐RD can manifest either in the pleura alone or in combination with other pulmonary symptoms. Choi and colleagues [10] described a 48‐year‐old patient with IgG4‐RD who presented with thrombophlebitis and a large pleural effusion. The sole thoracic feature in this patient was a pleural effusion. Yamashita et al. described a patient with a right‐sided effusion and lung parenchymal lesions [11]. Pleural effusion can occur with or without extrathoracic manifestations in patients with IgG4‐RD [12].

We considered other causes of peripheral blood eosinophilia in this case. Previous research has demonstrated that immune‐mediated pathways, activated by both autoimmune and viral pathogens, may play a role in the fibroinflammatory process. Zhang et al. showed that blood eosinophilia in IgG4‐RD was not influenced by allergies [13]. Numerous investigations have demonstrated that the peripheral blood eosinophil proportion heightens in the context of IgG4‐related pleural effusions [14]. Our patient had a documented history of asthma with symptoms that were well controlled, and he had mildly elevated peripheral eosinophils probably unrelated to asthma. Some reports have demonstrated the coexistence of asthma and IgG4‐RD [15].

The diagnosis of IgG4‐RD is established by international consensus, and histopathological criteria are the current gold standard [4]. In our patient, the presence of significant lymphoplasmacytic infiltration and obliterative phlebitis was contributory to the diagnosis. Moreover, the presence of > 10 IgG4‐positive plasma cells per high‐power field, in addition to an IgG4:IgG‐positive plasma cell ratio of greater than 40%, clinched the diagnosis. However, the presence of storiform fibrosis may vary. In general, it is not advisable to diagnose IgG4‐RD exclusively based on biopsy results. The involvement of lung in IgG4‐RD can occur as an isolated phenomenon or in conjunction with other systemic disorders, presenting a formidable diagnostic and therapeutic challenge, particularly in the absence of extensive long‐term data [16]. According to the diagnostic criteria, our patient falls into the “definitive” IgG4 disease category [17].

The treatment approach for pleural effusion is contingent on symptom severity. For patients with IgG4‐RD with pleural involvement and no multisystem disease, a benign prognosis is typically expected. Careful monitoring is usually sufficient in cases of minor and asymptomatic effusions [18]. While spontaneous recovery may occur, the condition frequently recurs without treatment. Systemic glucocorticoid therapy is recommended for individuals with symptoms or significant effusion, after excluding parasitic infections, connective tissue diseases and lymphoma, as was done in our patient. The suggested therapy involves the administration of prednisolone at an initial dosage of 0.5–0.6 mg/kg/day given for 2–4 weeks. This dose is then gradually reduced in subsequent months based on the progress of symptoms and laboratory and imaging characteristics to a maintenance level of 2.5–5 mg/day over 2–3 months. Comprehensive characterization and prediction of the natural course and outcomes have not been adequately specified. Most patients initially exhibited a positive response to glucocorticoid medication; however, relapses were observed once the therapy was stopped [19, 20]. In cases of relapsed IgG4‐RD, restarting or increasing the steroid dosage proves effective. The addition of immunomodulatory agents like azathioprine are deemed appropriate. New therapeutic strategies aimed at targeting B cells and/or CD4+ cytotoxic T lymphocytes (CTLs) are under investigation. B cell depletion using rituximab, an anti‐CD20 antibody, has shown effectiveness in patients who did not respond to immunomodulatory therapies [20, 21].

In conclusion, we have reported a rare case of IgG4‐RD presenting with EPE and minimal lung parenchymal involvement. Pleural effusion is uncommon in IgG4‐RD, which in turn is an uncommon cause of exudative pleural effusion. Also, IgG4‐RD is an extremely rare cause of EPE. To our knowledge, this is the first report of IgG4‐related EPE from India.

## Author Contributions

All authors were actively involved in the case report drafting and modification. Dr. Ancy Elsa Thomas and Dr. Balamugesh Thangakunam did the conceptualization, literature search, and manuscript writing. Dr. Balamugesh Thangakunam, Dr. Thomas Kodiatte, and Dr. Devasahayam Jesudas Christopher were involved in the design, manuscript review, and editing. The authors reviewed and approved the final paper.

## Ethics Statement

Written informed consent was obtained from the patient to publish this case report and the accompanying images.

## Conflicts of Interest

The authors declare no conflicts of interest.

## Data Availability

The data that support the findings of this study are available on request from the corresponding author. The data are not publicly available due to privacy or ethical restrictions.

## References

[1] D. J. Christopher , R. Gupta , B. Thangakunam , et al., “Pleural Effusion Guidelines From ICS and NCCP Section 1: Basic Principles, Laboratory Tests and Pleural Procedures,” Lung India 41, no. 3 (2024): 230–248, 10.4103/lungindia.lungindia_33_24. 38704658 PMC11093145

[2] P. Brito‐Zerón , M. Ramos‐Casals , X. Bosch , and J. H. Stone , “The Clinical Spectrum of IgG4‐Related Disease,” Autoimmunity Reviews 13, no. 12 (2014): 1203–1210, 10.1016/j.autrev.2014.08.013. 25151972

[3] Y. Fei , J. Shi , W. Lin , et al., “Intrathoracic Involvements of Immunoglobulin G4‐Related Sclerosing Disease,” Medicine 94, no. 50 (2015): e2150, 10.1097/md.0000000000002150. 26683924 PMC5058896

[4] V. Deshpande , Y. Zen , J. K. Chan , et al., “Consensus Statement on the Pathology of IgG4‐Related Disease,” Modern Pathology 25 (2012): 1181–1192, 10.1038/modpathol.2012.72. 22596100

[5] Y. Oba and T. Abu‐Salah , “The Prevalence and Diagnostic Significance of Eosinophilic Pleural Effusions: A Meta‐Analysis and Systematic Review,” Respiration 83 (2012): 198–208, 10.1159/000327200. 21576924

[6] Y. Murata , K. Aoe , and Y. Mimura , “Pleural Effusion Related to IgG4,” Current Opinion in Pulmonary Medicine 25 (2019): 384–390, 10.1097/MCP.0000000000000581. 30883447 PMC6613714

[7] Z. S. Wallace , V. Deshpande , H. Mattoo , et al., “IgG4‐ Related Disease: Clinical and Laboratory Features in One Hundred Twenty‐Five Patients,” Arthritis & Rheumatology 67 (2015): 2466–2475, 10.1002/art.39205. 25988916 PMC4621270

[8] I. Kalomenidis and R. W. Light , “Pathogenesis of the Eosinophilic Pleural Effusions,” Current Opinion in Pulmonary Medicine 10 (2004): 289–293, 10.1097/01.mcp.0000127902.37822.13. 15220754

[9] L. Wang , J. Di , J. Huang , and C. Guo , “IgG4‐Related Eosinophilic Pleural Effusion: A Case Report,” BMC Geriatrics 23, no. 1 (2023): 33, 10.1186/s12877-022-03594-3. 36658508 PMC9854229

[10] J. H. Choi , J. K. Sim , J. Y. Oh , et al., “A Case of IgG4‐Related Disease Presenting as Massive Pleural Effusion and Thrombophlebitis,” Tuberculosis and Respiratory Diseases (Seoul) 76 (2014): 179–183, 10.4046/trd.2014.76.4.179. PMC4021266 24851132

[11] K. Yamashita , H. Haga , Y. Kobashi , et al., “Lung Involvement in IgG4‐Related Lymphoplasmacytic Vasculitis and Interstitial Fibrosis: Report of 3 Cases and Review of the Literature,” American Journal of Surgical Pathology 32 (2008): 1620–1626, 10.1097/PAS.0b013e318172622f. 18753944

[12] C. Dragos , C. Joseph , H. Elwell , M. Dey , and K. Kouranloo , “Pulmonary Manifestations, Treatments and Outcomes of IgG4‐Related Disease–a Systematic Literature Review,” Rheumatology International 44, no. 10 (2024): 1875–1886, 10.1007/s00296-024-05611-7. 38769126 PMC11393110

[13] X. Zhang , P. Zhang , J. Li , et al., “Different Clinical Patterns of IgG4‐RD Patients With and Without Eosinophilia,” Scientific Reports 11 (2019): 16483, 10.1038/s41598-019-52847-6. PMC6848131 31712579

[14] S. Kasashima , A. Kawashima , Y. Zen , et al., “Upregulated Interleukins (IL‐6, IL‐10, and IL‐13) in Immunoglobulin G4‐Related Aortic Aneurysm Patients,” Journal of Vascular Surgery 67 (2018): 1248–1262, 10.1016/j.jvs.2016.12.140. 28434701

[15] Y. S. Lee , H. J. Cho , H. S. Yoo , Y. S. Shin , and H. S. Park , “A Case of IgG4‐Related Disease With Bronchial Asthma and Chronic Rhinosinusitis in Korea,” Journal of Korean Medical Science 29 (2014): 599–603, 10.3346/jkms.2014.29.4.599. 24753711 PMC3991807

[16] J. P. Corcoran , E. L. Culver , R. M. Anstey , et al., “Thoracic Involvement in IgG4‐Related Disease in a UK‐Based Patient Cohort,” Respiratory Medicine 132 (2017): 117–121, 10.1016/j.rmed.2017.10.005. 29229083

[17] A. Hegde , R. Upreti , S. Prashant , V. Gupta , J. Anurag , and U. Kovilapu , “An Unusual Presentation of IgG4‐Related Disease,” Indian Journal of Rheumatology 15, no. 3 (2020): 229, 10.4103/injr.injr_12_20.

[18] A. Khosroshahi , Z. S. Wallace , J. L. Crowe , et al., “International Consensus Guidance Statement on the Management and Treatment of IgG4‐Related Disease,” Arthritis & Rheumatology 67 (2015): 1688–1699, 10.1002/art.39132. 25809420

[19] Y. Masaki , H. Shimizu , T. Sato Nakamura , et al., “IgG4‐Related Disease: Diagnostic Methods and Therapeutic Strategies in Japan,” Journal of Clinical and Experimental Hematopathology 54, no. 2 (2014): 95–101, 10.3960/jslrt.54.95. 25318941

[20] T. Kamisawa and K. Okazaki , “Diagnosis and Treatment of IgG4‐Related Disease,” Current Topics in Microbiology and Immunology 401 (2017): 19–33, 10.1007/82_2016_36. 28197739

[21] C. A. Perugino and J. H. Stone , “IgG4‐Related Disease: An Update on Pathophysiology and Implications for Clinical Care,” Nature Reviews Rheumatology 16 (2020): 702–714, 10.1038/s41584-020-0500-7. 32939060

